# Experimental Animal Models of Arteriovenous Malformation: A Review

**DOI:** 10.3390/vetsci2020097

**Published:** 2015-06-19

**Authors:** Jude Amal Raj, Marcus Stoodley

**Affiliations:** The Australian School of Advanced Medicine, Macquarie University, NSW 2109, Australia; E-Mail: jude.amalraj@mq.edu.au

**Keywords:** cerebral arteriovenous malformations, animal models

## Abstract

Arteriovenous malformations (AVMs) are congenital lesions that cause brain haemorrhage in children and young adults. Current treatment modalities include surgery, radiosurgery and embolization. These treatments are generally effective only for small AVMs. Over one third of AVMs cannot be treated safely and effectively with existing options. Several animal models have been developed with the aims of understanding AVM pathophysiology and improving treatment. No animal model perfectly mimics a human AVM. Each model has limitations and advantages. Models contribute to the understanding of AVMs and hopefully to the development of improved therapies. This paper reviews animal models of AVMs and their advantages and disadvantages.

## 1. Introduction

Cerebral arteriovenous malformations (AVMs) are abnormal connections between arteries and veins that lead to the formation of a tangled collection of vessels referred to as a “nidus” [1,2,3]. The high-pressure shunt of arterial blood flowing through the fragile vessels in the nidus and the draining veins can cause intracranial haemorrhage resulting in death or disability [4,5]. The prevalence of this condition is 0.01%–0.5% of the population with presentation common in children and young adults [2,6]. In addition to haemorrhage, AVMs can present with epileptic seizures, chronic headaches, migraines and ischaemic neurologic deficits [7,8,9,10].

Current treatment options include surgical resection, endovascular occlusion and radiosurgery, or combinations of these [11]. Surgery is generally limited to small AVMs that are superficially located [12,13,14]. Large AVMs (more than 3 cm diameter) and those located in critical regions such as the thalamus and basal ganglia remain a challenge for effective treatment: There is a higher risk of morbidity following surgery in such cases [11,15,16]. Radiosurgery, although attractive as a relatively non-invasive treatment, is often not as effective as surgery [17,18]. There is also a 2–3 year delay to AVM occlusion after radiosurgery treatment and a success rate of approximately only 80% even for small lesions [14,19,20,21].

There is an urgent need for a new treatment for the over one third of AVMs that cannot be safely and effectively treated with current protocols [21,22]. Animal models are required to investigate the pathogenesis of AVM formation and their biological and haemodynamic characteristics. Development of a new effective treatment for AVMs is also likely to depend on the use of appropriate animal models. Improving responses of AVM tissue to radiation, and developing adjuvants such as radiosensitisers, could be best achieved using animal models. Potential new biological therapies such as gene therapy or vascular targeting to induce AVM thrombosis will require animal models for developing and trialling the therapeutic agents. Refining endovascular techniques is a further field where animal models are extremely useful.

Many animal models have been developed in the last three decades. Each has advantages and disadvantages and there is no model that is suitable for all purposes. This review discusses each of the research settings where models are used and discusses the advantages and disadvantages of each model. Standardising animal model use for each research indication would hopefully lead to uniformity in research methods and more efficient progress towards the goal of developing better treatments for AVM patients.

## 2. Study of AVM Haemodynamics

Replicating AVM haemodynamics is necessary for the study of AVM pathophysiology and refinement of endovascular techniques. Most models include an arteriovenous shunt and a nidus.

The rete mirabile in swine has been used as an AVM model for its network of microarteries that are interconnected and resemble an AVM nidus, at least angiographically [23]. However its arterial-to-arterial structure does not mimic that of a human AVM, which is arterial to venous [23]. This limitation was addressed by creating a short-term arteriovenous shunt between the rete and the internal carotid artery and the cavernous sinus [23]. Although this model has morphological resemblances to human AVMs, there are several adverse effects on the animals such as proptosis, chemosis and sub-conjunctival haemorrhage. The model lasts for a maximum period of only a week by which time the connections have occluded spontaneously. As with most AVM models, the rete mirabile model is not inside the brain. The model however is particularly useful for refining endovascular techniques and trialling new embolic agents [23].

In an attempt to simplify the creation of the model, Massoud *et al.*, created an AVM model in the neck of swine, thus avoiding the adverse effects of the rete-cavernous fistula model. In order to increase blood flow in the fistula, three arteries are occluded in the neck region. This is followed by a side-to-side anastomosis forming a carotid-jugular fistula [24].

**Figure 1:**
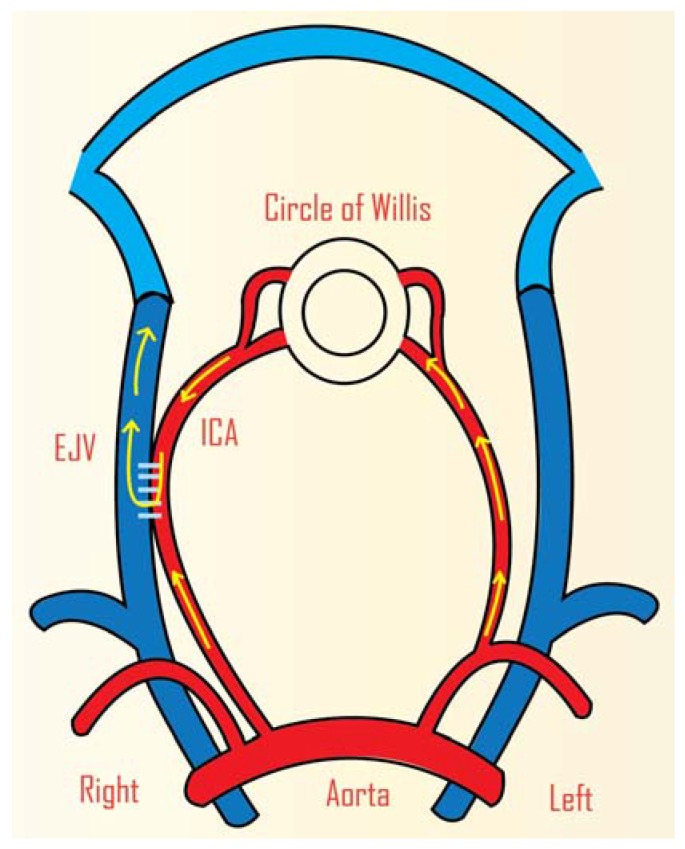
Animal model of cerebral AVM in sheep. Side-to-side anastomosis was carried out between the CCA and EJV (Right). The arrows show the direction of the circulatory flow after anastomosis. ICA: common carotid artery, EJV: external jugular vein. (Adapted from Qian *et al.*, 1990) [25].

This model has been criticised for its use of microcatheter and detachable balloons, which are very expensive [25]. Qian *et al.* created a similar model in sheep, but did not occlude the arterial branches (Figure 1). The sheep rete mirabile is quite different to that in swine. The retia appear as two units, connected by bridging vessels in sheep and as one in swine. This proves to be an advantage for sheep over swine where it gives the possibility for researchers to study multiple AVMs [25].

Swine are preferred for their large neck vessels, which allow for faster and easier fistula creation. There are models in which external arteries need not be occluded [26,27]. An end-to-end anastomosis instead of a side-to-side anastomosis shows an increase in blood flow in the rete mirabile. Spontaneous occlusion does not occur as in previous models, which means these could be used for research that involves a longer duration [23,24,26,27]. Additional advantages with swine model are that the swine coagulation system is similar to humans and they are inexpensive [24].

**Figure 2:**
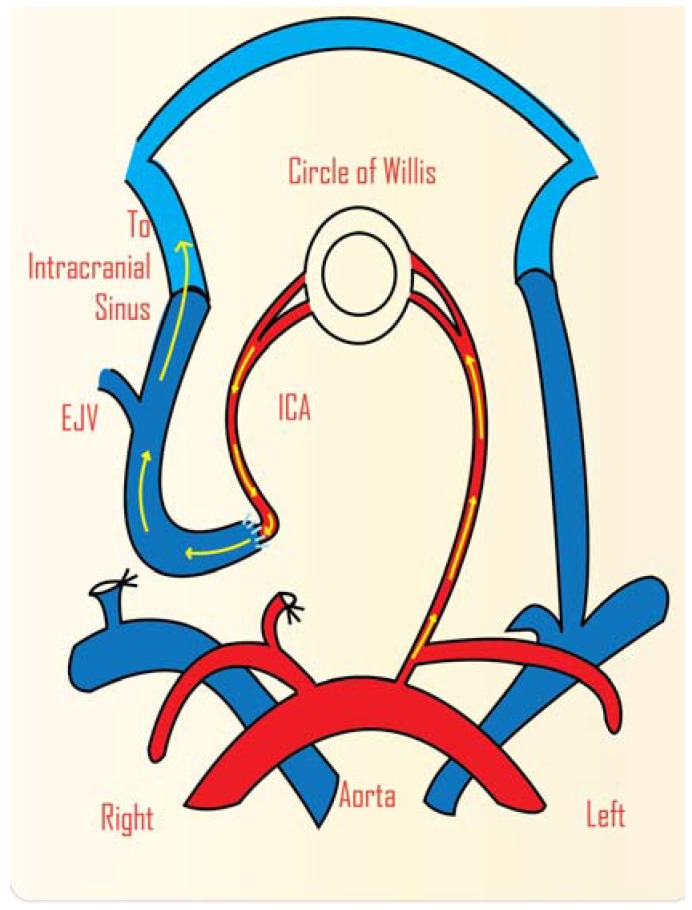
Schematic representation of animal model of cerebral AVM in rat. Carotid jugular fistula model that involves an end-to-end anastomosis between the common carotid artery and external jugular vein. ICA: internal carotid artery; EJV: external jugular vein. (Adapted from Morgan *et al.*, 1989) [32].

Haemodynamic models are also used to study perfusion pressure breakthrough, the role of venous hypertension and the role of thrombosis in AVMs. Rat models are often used for research in these areas [28,29]. Various haemodynamic arrangements have been used, although the model usually is located in the neck region by creating an anastomosis between the common carotid artery and external jugular vein [6,30]. In these models, the common carotid artery acts as the feeding artery and the external jugular vein acts as the arterialized vein. In one model, the anastomosis is between the rostral carotid artery and the caudal jugular vein. This arrangement results in intracranial hypoperfusion, analogous to the “steal” phenomenon around AVMs, and has been used to study the effects of chronic ischaemia [31,32]. The carotid jugular fistula model by Morgan *et al.* was used to study hypoperfusion. An end-to-end anastomosis was performed between the common carotid artery and the external jugular vein (Figure 2). The involvement of both the intracranial arterial and venous systems with the extracranial venous systems is a feature of this model [32].

**Figure 3:**
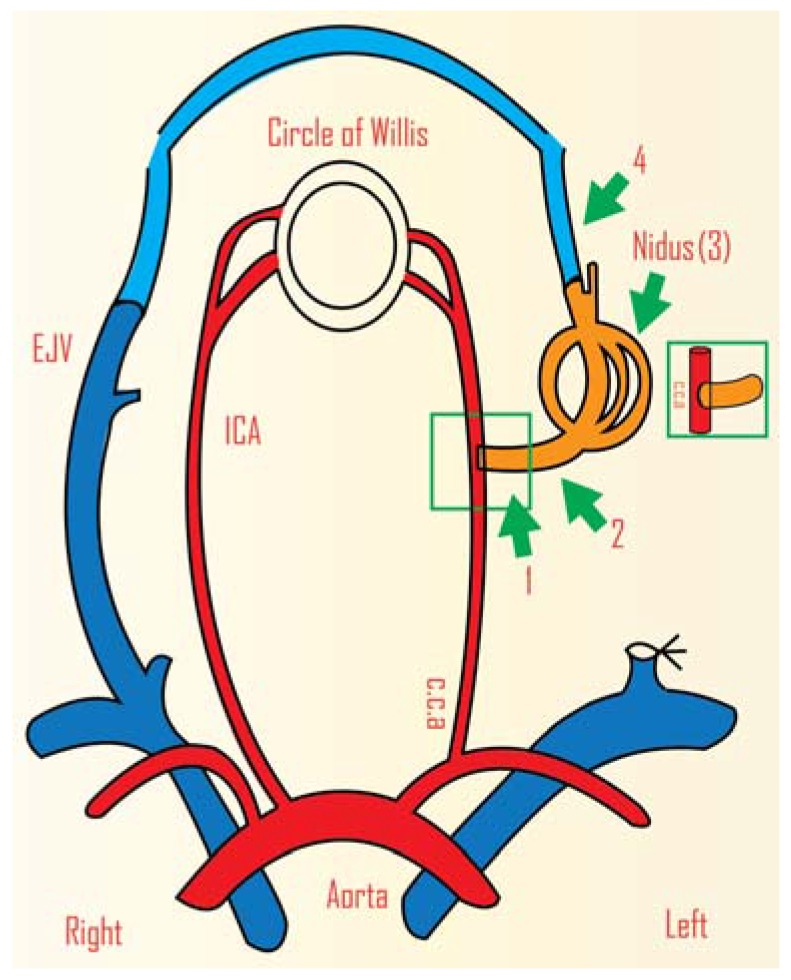
Schematic representation of an AVM model in rat. This is a carotid jugular, side-to-end anastomosis between the common carotid artery and external jugular vein. This is a very efficient model for radiosurgery related studies. CCA: common carotid artery, ICA: internal carotid artery, EJV: external jugular vein. Anastomosis (**1**) arterial feeder (**2**) nidus (**3**) draining vein (**4**).

In other models, the anastomosis is a side-to-end connection between the common carotid artery and the rostral external jugular vein. In this arrangement arterial blood flows through the external jugular vein and its branches (the “nidus”) and the outflow is then through the transverse sinus and the contralateral jugular vein (draining vein) (Figure 3) [33,34]. This model has been used to study the molecular and morphological changes in AVMs treated with radiosurgery [6,35]. Critically, the model produces an endothelial phenotype that resembles that of human AVMs; this is crucial when studying radiation effects because the endothelial response to radiation is different for different phenotypes. Blood flow has been shown to increase with time in this model, at least until 42 days post-surgery [6].

**Figure 4:**
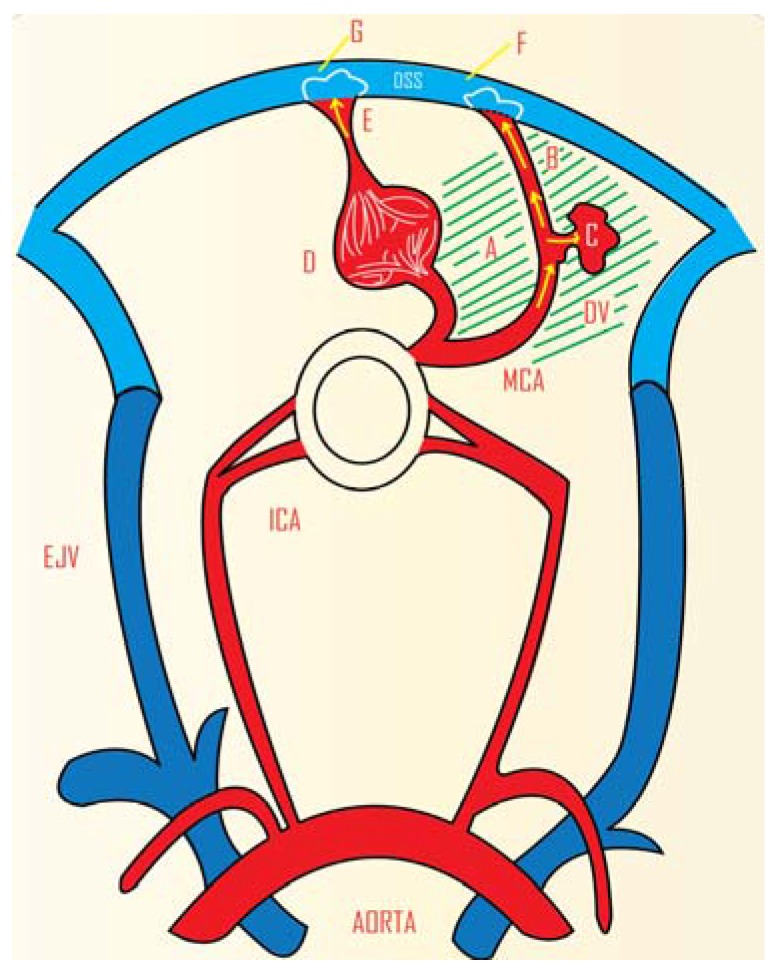
Animal model of cerebral AVM in beagle dogs. The dog model located intracranially has the benefit of the vascular capillary network around the AV shunt, resembling the human AVM more closely. DSS: Dorsal sagittal sinus MCA: Middle cerebral artery Area shaded with slanted lines: Relative ischaemia to stimulate angiogenesis. **A**: End-to-end anastomosis **B**: End-to-side anastomosis **C**: Muscle graft implanted in the ischaemic brain tissue and supplied by the bypass **D**: Nomal capillary network in the brain parenchyma **E**: Vein with normal drainage **F:** Arterialized blood **G**: Venous blood. (Adapted from Pietila *et al.*, 2000) [38].

In the many AVM models that claim to have a blood flow similar to what is observed in human AVMs, the model is usually extracranial in location and not intracranial [24,36,37]. In response to this, an AVM model was developed in beagle dogs (Figure 4) [38]. An end-to-end anastomosis between the superficial temporal artery and the middle cerebral artery and an end to side anastomosis between the superficial temporal artery and the dorsal sagittal sinus were carried out. A muscle graft supplied by the superficial temporal artery was fixed in the ischaemic region of the brain. Ischaemia was found to be aggravated in the arteriovenous fistula region [38]. An advantage of this model is its intracranial location. This model has not been reported in any subsequent investigations.

A model was developed by Numazawa *et al.* to study hypoperfusion and impaired CO_2_ reactivity. A femoral vein graft was used to connect a cortical branch of the middle cerebral artery and the superior sagittal sinus, thus creating an arteriovenous shunt. This model was shown to closely resemble the haemodynamics of AVM patients with hypoperfusion and decreased CO_2_ reactivity [39].

The rete mirabile is a suitable model for the development of endovascular techniques. Surgery is not required for this arterio-arterial system. However for studies involving the pathophysiology of AVMs and their haemodynamic properties, an arterio-venous shunt is required. This swine model can be used for this purpose, however the arteriovenous model in rats has an advantage in terms of simpler animal care and maintenance and is far more economical than swine and sheep.

## 3. Radiosurgery

Radiosurgery is one of the treatment options for AVMs [40,41,42]. A single, focussed high dose of radiation is delivered to a target volume of AVM tissue [42,43]. Free radicals react with the DNA causing damage [44,45]. This is followed by endothelial proliferation, smooth muscle cell proliferation and thrombosis, which lead to AVM obliteration [43]. The exact mechanism is still unknown. AVM animal models have therefore been developed to study the short-term effects of radiosurgery.

De Salles *et al.* treated eight swine with 20, 30, 40, 50, 60, 70, 80 and 90 Gy dosages. The rete mirabile was selected as the target tissue. Neurologic dysfunctions, such as abnormal eye movement, were commonly observed in the animals. A decrease in vascularity was observed in animals that received 50 Gy or more dosage and complete obliteration of the rete mirabile was observed in the animal that received 90 Gy [46]. The rete mirabile, which is located in the cavernous sinus, is an arterioarterial system unlike an AVM that is an arteriovenous system [47,48,49]. This means that that model lacks the high-pressure blood flow in arterialized veins typical for an AVM.

Jahan *et al.* used the model by Massoud *et al.* to study the effects of radiosurgery on a swine model where a carotid-jugular fistula was created in the neck [50,51]. The preferred dosage was 40 Gy. Decreased vascularity was observed in treated animals when compared to those not treated at a three-month time point [50]. Histopathological changes post-radiation were studied. The study showed expressions of Type IV collagen to be very similar to those in resected specimens post-radiation from humans [50].

Radiosurgery is shown to cause vascular obliteration in several models. Intimal hyperplasia, necrosis, oedema and gliosis have been observed in the tissues surrounding the target volume [46]. In the rete mirabile model for instance, these could be pointed to as the main cause for several neurological deficits such as hemiparesis, loss of coordination, disruption in vision and seizures [46]. Mut *et al.* studied a carotid jugular fistula model in a rat to see the effects of radiosurgery in the tissues surrounding the target volume for a dosage of 25 Gy [43]. DNA strand breaks and the level of free radicals were analysed. There was significant apoptosis in irradiated groups irrespective of the presence of the fistula [43].

Great care is taken in the complete obliteration of AVMs during surgical excision. A small amount of tissue left behind is capable of AVM angiogenesis. This applies to the radiosurgery treatment modality as well. AVM angiogenesis after radiosurgery is highly dose dependent according to the corneal angiogenesis model study [52]. Resected AVM specimens from humans were implanted in the corneal tissues of rats. Dosages of 1.5 Gy, 3 Gy, 15 Gy and 30 Gy were administered to the animals. The latter two dosage groups showed a greater anti-angiogenesis effect than the former two [52].

Another major application radiosurgery models have is in the search for a potential vascular target for inducing thrombosis. In the field of cancer, vascular targeting has been successful in inducing thrombosis due to the molecular differences that distinguish tumor vessels from normal vessels [33]. This however requires the AVM vessels to differ significantly from normal vessels. Tu *et al.* have reported increased levels of ICAM, VCAM and E selectin in human AVM endothelium when compared to the endothelium in normal vessels, however the changes were not sufficiently discriminating to be used as a vascular target [53].

The carotid jugular anastomosis rat model was to see if radiosurgery induced molecular changes sufficient to discriminate them from normal vessels [53]. In this model, the external jugular vein was anastomosed to the common carotid artery. After six weeks when the fistulae were considered mature, AVM niduses were treated with radiosurgery (25 Gy). In this work, radiosurgery was given not as a treatment for the AVM but rather as a priming technique to explore the endothelial molecular changes that would come about as a result of radiosurgery [54]. Subsequent studies have used the model to demonstrate induced thrombosis with vascular targeting [33,35].

Swine and rat models are highly suitable to study the effects and efficacy of radiosurgery. The swine model is resource intensive and suffers from the fact that the “nidus” (rete mirabile) is purely arterial. The rat model is more practical and consists of arterialized veins that more closely resemble the molecular characteristics of human AVMs.

## 4. Genetic Studies

There is a growing interest in gene therapy to treat large AVMs and those located in regions such as the thalamus, basal ganglia and brainstem that cannot be treated by surgery [55,56,57,58]. For this to be successful, a family of molecules or a particular gene responsible for a certain characteristic feature such as narrowing of the arteries needs to be identified [58,59]. Once identified, that particular family of molecules or the gene may be overexpressed to remove the AVM successfully [58]. AVMs are typically sporadic [60]. However, 2% of AVMs are familial, including patients with hereditary haemorrhagic telangiectasia (HHT) or Rendu-Osler Weber syndrome [60,61,62]. In HHT, abnormal malformations in different organs such as the lung, liver, brain and spine occur as a result of mutations in the endoglin and ACVRL1 genes [63,64]. The causal factors for this genetic disorder are unknown [65]. This poses the need for HHT animal models to better understand the pathophysiology of HHT and also to develop new therapeutic modalities. Animal model studies have successfully been able to identify certain genetic mutations and risk factors [2,66]. The TGF-β genes are a good example of those associated with non-sporadic AVMs. The TGF-β genes go through a loss of function mutation in endoglin and activin like kinase giving rise to the HHT (1 and 2) mutation resulting in AVM formation [65,67,68].

In the first transgenic mouse model study by Satomi *et al.*, ten endoglin heterozygous mice (Eng^+/−^) were compared with 15 controls (Eng^+/+^) mice. AVMs are believed to develop in the mice that are heterozygous at the endoglin locus [69]. Only 30% showed abnormalities that closely resemble those present in human AVMs. However, no abnormalities were observed in any of the control animals. Changes such as decreased arteriole constriction and increased relative dilatation were also observed. More research is required to determine if these changes actually contribute to the formation of AVMs in HHT patients [70]. In another mouse model that was stimulated with VEGF (vascular endothelial growth factor), 89% of the Eng^+/−^ mice showed vascular abnormalities and none were observed in the controls [71]. The lung, liver and intestine showed dilation in some vessels with no external signs in one particular animal [70]. Another animal that had an ear telangiectasis showed an AVM nidus that resembled those in human and canine brain [70]. Despite the fact that Eng^+/−^ and Acvrl1^+/−^ mice are the best available HHT models at present, past studies do not show the HHT features at high frequency [69,72,73]. Although the two studies could be criticised as not being true AVM models and for their small sample size, they may contribute to research on the abnormal phenotype of AVMs in humans [66].

A transgenic arteriovenous fistula between the common carotid artery and external jugular vein with TAo from a donor mouse was created in 112 rats to investigate a possible gene therapy for AVMs. Grafting however would impose the challenge of not being able to investigate certain genetic changes in the fistula [58]. Limitations such as low lesion frequency in Eng^+/−^ mice and lack of the human AVM phenotype in currently existing models call for more work to be carried out in this area of study. Two HHT 1 animal models were created to study the developmental and adult onset of cerebral AVMs [74]. *Eng* conditional knockout mouse lines were obtained by crossing *Eng*^2fl/2fl^ with R26CreER, SM22α-Cre and LysM-Cre. The Eng^2fl/2fl^ were crossbred with SM22αCre to study the effects of embryonic *Eng* deletion on the post-natal development of AVMs [74]. Almost 90% from the group *Eng*^2fl/2fl^; SM22α-Cre showed the AVM phenotype. No lethality was observed until the fifth week [74]. In previous similar models involving a conditional knockout of the Alk1 gene, intracranial haemorrhage led to lethality by even post-natal day 5 and two weeks limiting the use of such models in studies related to new therapies [75,76]. The *Eng*^2fl/2fl^; R26CreER were given a dose of tamoxifen for three consecutive days to induce *Eng* deletion. Those mice that were additionally injected with adeno-associated viral vector expressing vascular endothelial growth factor showed AVM phenotype in the form of lesions eight weeks after *Eng* deletion. No lethality was observed in the *Eng*^2fl/2fl^; R26CreER group upto two months post-tamoxifen treatment [74].

Transgenic animal studies are useful for investigation of gene changes. Any potential gene therapy would need to be trialled on such models. The major drawback with these models is in identifying AVMs in individual animals.

## 5. Conclusions

Animal models have been developed because naturally occurring AVMs in animals are quite rare. There is no animal model that is perfect with all the necessary characteristics. The choice of model will depend on the purpose of the research. The rete mirabile is suitable for angiographic studies, but the lack of arterialized veins makes the model unsuitable for studies of AVM biology. Models with an arteriovenous shunt are useful for studying the biology of arterialized veins and for investigating the effects of cerebral hypoperfusion. Investigations using radiosurgery should use a model with arterialized veins and an identifiable model nidus. Transgenic models are appropriate for research related to HHT and for genetic studies.
